# High expression of *myocyte enhancer factor 2C* (*MEF2C*) is associated with adverse-risk features and poor outcome in pediatric acute myeloid leukemia: a report from the Children’s Oncology Group

**DOI:** 10.1186/s13045-015-0215-4

**Published:** 2015-10-20

**Authors:** George S. Laszlo, Todd A. Alonzo, Chelsea J. Gudgeon, Kimberly H. Harrington, Alex Kentsis, Robert B. Gerbing, Yi-Cheng Wang, Rhonda E. Ries, Susana C. Raimondi, Betsy A. Hirsch, Alan S. Gamis, Soheil Meshinchi, Roland B. Walter

**Affiliations:** Clinical Research Division, Fred Hutchinson Cancer Research Center, 1100 Fairview Ave N, D2-190, Seattle, WA 98109-1024 USA; Department of Biostatistics, University of Southern California, Los Angeles, CA USA; Children’s Oncology Group, Monrovia, CA USA; Molecular Pharmacology and Chemistry Program, Sloan Kettering Institute, New York, NY USA; Department of Pediatrics, Memorial Sloan Kettering Cancer Center, New York, NY USA; Weill Medical College of Cornell University, New York, NY USA; Department of Pathology, St. Jude Children’s Research Hospital, Memphis, TN USA; Department of Laboratory Medicine and Pathology, University of Minnesota Cancer Center, Minneapolis, MN USA; Division of Hematology-Oncology, Children’s Mercy Hospitals and Clinics, Kansas City, MO USA; Department of Pediatrics, University of Washington, Seattle, WA USA; Department of Medicine, Division of Hematology, University of Washington, Seattle, WA USA; Department of Epidemiology, University of Washington, Seattle, WA USA

**Keywords:** AAML0531, Acute myeloid leukemia (AML), Adverse risk, Biomarker, Children’s Oncology Group (COG), Myocyte enhancer factor 2C (MEF2C), Pediatric, Transcription factor

## Abstract

**Background:**

Recent studies have identified *myocyte enhancer factor 2C* (*MEF2C*) as cooperating oncogene in acute myeloid leukemia (AML) and suggested a contribution to the aggressive nature of at least some subtypes of AML, raising the possibility that *MEF2C* could serve as marker of poor-risk AML and, therefore, have prognostic significance.

**Methods:**

To test this hypothesis, we retrospectively quantified *MEF2C* expression in pretreatment bone marrow specimens in participants of the AAML0531 trial by reverse-transcriptase polymerase chain reaction and correlated expression levels with disease characteristics and clinical outcome.

**Results:**

In all 751 available patient specimens, *MEF2C* messenger RNA (mRNA) was detectable and varied >3000-fold relative to *β-glucuronidase*. Patients with the highest relative *MEF2C* expression (4th quartile) less likely achieved a complete remission after one course of chemotherapy than the other patients (67 vs*.* 78 %, *P* = 0.005). They also had an inferior overall survival (*P* = 0.014; at 5 years 55 ± 8 vs. 67 ± 4 %), inferior event-free survival (*P* < 0.001; at 5 years 38 ± 7 vs. 54 ± 4 %), and higher relapse risk than patients within the lower 3 quartiles of *MEF2C* expression (*P* < 0.001; at 5 years 53 ± 9 vs. 35 ± 5 %). These differences were accounted for by lower prevalence of cytogenetically/molecularly defined *low-risk* disease (16 vs*.* 46 %, *P* < 0.001) and higher prevalence of *standard-risk* disease (68 vs. 42 %, *P* < 0.001) in patients with high *MEF2C* expression, suggesting that *MEF2C* cooperates with additional pathogenic abnormalities.

**Conclusions:**

High *MEF2C* expression identifies a subset of AML patients with adverse-risk disease features and poor outcome. With confirmation that high *MEF2C* mRNA expression leads to overexpression of MEF2C protein, these findings provide the rationale for therapeutic targeting of *MEF2C* transcriptional activation in AML.

## Background

Myocyte enhancer factor 2 (MEF2) proteins, composed of four family members in vertebrates, are transcription factors that were initially studied in the control of muscle development [1]. In particular, gene deletion studies in mice identified essential functions of *MEF2C* in cardiac myogenesis and right ventricular development [2]. However, subsequent studies have indicated that *MEF2C* plays a much broader biological role and is involved in the function and generation of tissues other than cardiac and skeletal muscle, including bone development and osteoclast-mediated bone resorption, neuronal development, and craniofacial and melanocyte development [3].

Increasing evidence also suggests an important role of *MEF2C* in the normal hematopoietic system, particularly for the production of immature and mature lymphoid cells and as a modulator of the cell fate decision between monocyte and granulocyte differentiation [3–6]. This is indicated by genetic studies in mice showing that *Mef2c* deficiency is associated with reduced levels of monocytes in response to cytokines [4] as well as profound defects in the production of B cells, T cells, natural killer cells, and common lymphoid progenitor cells, as well as enhanced myeloid output [5]. In turn, constitutive expression of *Mef2c* in the bone marrow results in increased monopoiesis at the expense of granulopoiesis [4]. In human acute myeloid leukemia (AML) cell line models, 1,25-dihydroxyvitamin D3 induces monocytic differentiation and CD14 expression, an effect that is mediated through activation of *MEF2C* signaling via regulation of CCAAT-/enhancer-binding protein alpha (CEBPA) [6]. Consistent with these central functions, *MEF2C* has been found to be aberrantly expressed in subsets of T cell acute lymphoblastic leukemia (T-ALL) and in early thymocyte precursor (ETP) T-ALL in particular, an aggressive leukemia that tends to be refractory to chemotherapy and shares genetic features with AML [7–10]. In AML, *MEF2C* has been found to be overexpressed in distinct molecular subsets of adult onset AML, including mixed lineage leukemia (*MLL*) gene-rearranged and ectropic virus integration site 1 (*EVI1*)-overexpressing leukemias [11]. Models of both MLL and EVI1 leukemias have been, and continue to be, instrumental in our understanding of fundamental principles of leukemogenesis and the identification of pathways that confer tumor aggressiveness and resistance to chemotherapy [12–21]. Functional studies using mouse leukemia models demonstrate that *Mef2c* is a potent oncogene, causing fully penetrant AML in cooperation with *SOX4* [11, 22, 23]. In addition, *Mef2c* is required for the growth of mouse leukemias induced by *MLL-AF9* [11].

Together, these studies suggest that *MEF2C* participates in key molecular mechanisms of AML pathogenesis and could serve as a marker of poor-risk AML and, therefore, have prognostic significance. Here, we tested this hypothesis by retrospectively quantifying *MEF2C* expression in pretreatment bone marrow specimens and by associating *MEF2C* expression level with disease characteristics and outcome in participants of the Children’s Oncology Group (COG) AML protocol, AAML0531 (NCT00372593). AAML0531 was a multicenter, randomized phase 3 study, which found that the addition of gemtuzumab ozogamicin to intensive chemotherapy improved the event-free survival (EFS) through reduction of the relapse risk (RR) relative to intensive chemotherapy alone in patients aged <30 years with newly diagnosed de novo non-APL AML, excluding those with bone marrow failure syndromes, juvenile myelomonocytic leukemia, or Down syndrome (if ≤3 years of age) between 2006 and 2010 [24].

## Results

### Identification of *MEF2C* expression as predictive biomarker in participants of AAML0531

**Fig. 1:**
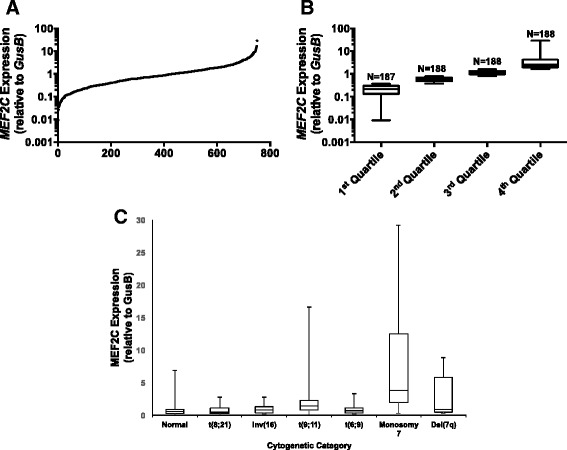
*MEF2C* expression in AAML0531. Quantitative expression of *MEF2C *relative to beta glucuronidase (*GUSB*) in diagnostic bone marrow specimens from the 751 patients who were included in this study. **a** Relative *MEF2C* expression across the entire study cohort. **b** Distribution of relative *MEF2C* expression across quartiles of *MEF2C* expression. **c** Distribution of relative *MEF2C* expression across individual cytogenetic categories

Among the 1022 eligible patients enrolled in AAML0531, 980 (96 %) consented to have diagnostic bone marrow specimens banked for future cancer research. At the time this research was conducted, RNA was available from 765 patients. Fourteen samples were excluded because of inadequate RNA as determined by low β-glucuronidase (GUSB) expression (Ct > 33.09). The remaining 751 patients (77 %) were used for quantitation of *MEF2C* expression levels. In all of these specimens, *MEF2C* mRNA was detectable and varied >3000-fold relative to *GUSB* mRNA (0.0091–29.1272 [median 0.7978]; Fig. 1).

### Association between *MEF2C* expression and clinical outcome

Studying the relationship between *MEF2C* expression and clinical outcome, we initially analyzed patient outcomes per quartile of *MEF2C* expression and noticed that the 188 patients with the highest relative *MEF2C* expression (4th quartile, corresponding to an expression of ≥1.66 relative to *GUSB*) fared worse than the 563 patients in the 1st, 2nd, or 3rd quartiles of *MEF2C* expression, respectively, with little difference between the first 3 quartiles. We therefore subsequently compared patients with the highest relative *MEF2C* expression (4th quartile) to patients with lower expression (1st to 3rd quartile).

**Fig. 2:**
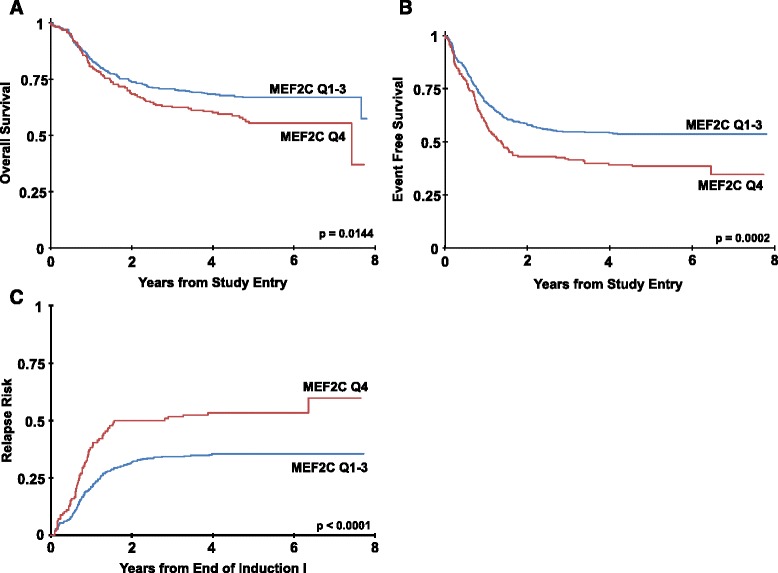
Clinical outcome in patients with high and low *MEF2C* expression in AAML0531. Estimates of the probability of OS (**a**), EFS (**b**), and RR (**c**) in patients with high (Q4) vs. low (Q1–3) relative *MEF2C* expression

Analyzing responses to initial chemotherapy, we found that patients with high relative *MEF2C* expression were statistically significantly less likely to have achieved a complete remission (CR) after one course of chemotherapy than patients with lower *MEF2C* expression (67 vs. 78 %, *P* = 0.005) and tended to be more likely to have flow cytometrically detectable minimal residual disease (MRD) at the end of the first induction course (33 vs. 27 %, *P* = 0.132). Some patients with high *MEF2C* expression were able to achieve remission with re-induction therapy, and the proportion of patients with high *MEF2C* expression in CR after two courses of induction chemotherapy approached that of patients with low *MEF2C* expression (86 vs. 90 %, *P* = 0.102). We subsequently evaluated how *MEF2C* expression related to parameters of long-term outcome and found that patients with the highest *MEF2C* expression had an inferior overall survival (OS; *P* = 0.014; at 5 years 55 ± 8 vs. 67 ± 4 %), inferior EFS (*P* < 0.001; at 5 years 38 ± 7 vs. 54 ± 4 %), and higher RR than the patients within the lower 3 quartiles of *MEF2C* expression (*P* < 0.001; at 5 years 53 ± 9 vs. 35 ± 5 %; Table 1 and Fig. 2a–c). Of note, exploratory multiple cutpoint analyses for OS and EFS indicated that the most statistically significant results were centered around the Q4 cutpoint region, supporting our approach of comparing patients with the highest quartile of relative *MEF2C* expression with those having lower relative *MEF2C* expression (data not shown).

**Table 1:** Comparison of treatment responses of patients with low (Q1–3) vs. high (Q4) *MEF2C* expression

Outcome at 5 years	Relative *MEF2C* expression				*P* value*
	Low (Q1–3)	High (Q4)	Hazard ratio^a^	95 % confidence interval	
All patients	*n* = 563	*n* = 188			
OS	67 ± 4 %	55 ± 8 %	1.385	1.07–1.80	0.014
EFS	54 ± 4 %	38 ± 7 %	1.510	1.21–1.88	<0.001
RR	35 ± 5 %	53 ± 9 %	1.813	1.36–2.42	<0.001
Low-risk patients	*n* = 255	*n* = 29			
OS	81 ± 5 %	76 ± 20 %	1.433	0.43–4.82	0.561
EFS	69 ± 6 %	51 ± 20 %	1.597	0.90–2.82	0.104
RR	23 ± 6 %	49 ± 20 %	2.290	1.26–4.17	0.011
Standard-risk patients	*n* = 230	*n* = 123			
OS	55 ± 7 %	55 ± 9 %	0.992	0.71–1.38	0.961
EFS	42 ± 7 %	38 ± 9 %	1.143	0.86–1.52	0.356
RR	49 ± 8 %	55 ± 11 %	1.270	0.88–1.83	0.201
High-risk patients	n = 69	n = 30			
OS	52 ± 12 %	37 ± 10 %	1.452	0.81–2.59	0.204
EFS	31 ± 11 %	29 ± 17 %	1.155	0.70–1.92	0.576
RR	46 ± 16 %	47 ± 27 %	1.381	0.59–3.22	0.446

*Log-rank *P* value

^a^Estimates from Weibull parametric models

We next performed subgroup analyses to investigate the association between *MEF2C* expression and outcome in specific risk groups; these studies were of exploratory nature since our ability to perform these analyses was relatively limited given the sample size of the individual risk groups. As summarized in Table 1, the association between high *MEF2C* expression and increased risk of relapse and, consequently, lower EFS was particularly apparent in the subset of cytogenetically/molecularly defined *low-risk* patients, whereas no strong trend was seen in patients with *standard-risk* or *high-risk* patients.

### Association between *MEF2C* expression and characteristics of study population

To investigate associations between relative *MEF2C* expression and demographics, baseline laboratory findings, and pretreatment characteristics of the study cohort, we compared patients with high *MEF2C* expression (4th quartile) with those having low *MEF2C* expression (1st to 3rd quartile). As summarized in Table 2, patients with high *MEF2C* expression were younger (*P* < 0.001) and more likely presented with hepatomegaly (*P* = 0.006) or splenomegaly (*P* < 0.001). They also had a slightly but statistically significantly higher percentage of bone marrow blast at diagnosis. In contrast, there was no statistically significant difference in gender distribution, white blood cell (WBC) count, or hemoglobin between patients with high and low *MEF2C* expression. Importantly, however, *MEF2C* expression was strongly associated with cytogenetic and molecular abnormalities. Specifically, patients with high *MEF2C* expression less likely had CBF translocations (inv(16): *P* = 0.007 and t(8;21): *P* < 0.001) or normal karyotype AML (*P* < 0.001); conversely, they were more likely to have leukemias with monosomy 7 (*P* < 0.001) and abnormalities involving 11q23 (*P* < 0.001). Furthermore, patients with high *MEF2C* less likely had a *FLT3/ITD* (*P* = 0.018) or a mutation in either *NPM1* (*P* = 0.010) or *CEBPA* (*P* = 0.002). Consistently, patients with high *MEF2C* expression less likely had *low-risk* disease (16 vs. 46 %, *P* < 0.001) and more likely had *standard-risk* disease (68 vs. 42 %, *P* < 0.001) than those with lower *MEF2C* expression (Table 2).

**Table 2:** Comparison of baseline characteristics of patients with low (Q1–3) vs. high (Q4) *MEF2C* expression

Patient characteristics	Relative *MEF2C* expression		*P* value
	Low (Q1–3)	High (Q4)	
	*n* = 563	*n* = 188	
Median age, years (range)	10.55 (0.01–29.8)	6.9 (0.06–19.8)	<0.001
Male sex, *n* (%)	279 (50 %)	95 (51 %)	0.817
WBC (×10^3^/μL), median (range)	30.7 (0.2–827.2)	20.9 (0.5–519.0)	0.160
Median bone marrow blasts, %	67.5 (0–100)	71 (3–100)	0.038
Platelet count (×10^3^/μL), median (range)	48 (4–556)	48 (1–11,177)	0.484
Hemoglobin (g/dL), median (range)	8.1 (2.3–17.0)	8.1 (1.8–17.0)	0.684
Cytogenetics, *n* (%)			
Normal	142 (26 %)	21 (12 %)	<0.001
t(8;21)(q22;q22)	101 (18 %)	10 (6 %)	<0.001
inv(16)/t(16;16)(p13.1;q22)	78 (14 %)	12 (7 %)	0.007
t(9;11)(p22;q23) or other abn 11q23	87 (16 %)	67 (37 %)	<0.001
t(6;9)(p23;q34)	10 (2 %)	1 (1 %)	0.309
Monosomy 7	3 (1 %)	11 (6 %)	<0.001
Del7q	4 (1 %)	2 (1 %)	0.642
−5/5q−	6 (2 %)	4 (2 %)	0.275
Trisomy 8	28 (5 %)	19 (10 %)	0.011
Other	89 (16 %)	34 (19 %)	0.428
Unknown	15	7	
Risk group, *n* (%)			
Standard	230 (42 %)	123 (68 %)	<0.001
Low	255 (46 %)	29 (16 %)	<0.001
High	69 (12 %)	30 (16 %)	0.170
Unknown	9	6	
Molecular alterations, %			
*FLT3/ITD*	18 %	10 %	0.018
*NPM1* mutation	9 %	3 %	0.010
*CEBPA* mutation	7 %	1 %	0.002
*WT1* mutation	6 %	5 %	0.688
Hepatomegaly, %	25 %	36 %	0.006
Splenomegaly, %	25 %	39 %	<0.001
Extramedullary disease, %	15 %	11 %	0.196
CNS disease, %	6 %	9 %	0.239
Chloroma, %	15 %	12 %	0.496
Treatment arm, *n* (%)			0.689
Arm A—no GO	281 (50 %)	97 (52 %)	
Arm B—with GO	282 (50 %)	91 (48 %)	

### *MEF2C* expression as an independent predictive factor

Finally, we evaluated the potential role of *MEF2C* expression as an independent predictor of OS, EFS, and RR in regression models (Table 3). Given the strong association between disease risk and *MEF2C* expression, one might attribute the worse outcome for patients with high *MEF2C* expression to the lower prevalence of leukemias with more favorable prognoses in this subgroup. Indeed, after adjustment for disease risk, age, FAB category, and treatment arm, high *MEF2C* expression was no longer statistically significantly associated with inferior OS (HR = 0.99 [0.72–1.36], *P* = 0.929), inferior EFS (HR = 1.14 [0.86–1.49], *P* = 0.365), or higher RR (HR = 1.32 [0.91–1.92], *P* = 0.137; Table 3).

**Table 3:** Univariate and multivariate regression models of OS, EFS, and RR

	OS						EFS							RR					
	*n*	HR			95 % CI	*P* value	*n*	HR	95 % CI			*P* value		*n*	HR	95 % CI			*P* value
Univariable model																			
*MEF2C* Expression																			
Low (Q1–3)	563	1					563	1						430	1				
High (Q4)	188	1.385			1.07–1.80	0.014	188	1.510	1.21–1.88			<0.001		126	1.813	1.36–2.42			<0.001
Disease risk^a^																			
Standard-risk	353	1					353	1						254	1				
Low-risk	284	0.351			0.26–0.48	<0.001	284	0.440	0.34–0.56			<0.001		234	0.410	0.30–0.56			<0.001
High-risk	99	1.234			0.90–1.69	0.193	99	1.377	1.05–1.81			0.022		57	0.886	0.59–1.34			0.567
FAB Class																			
Not M0	624	1					624	1						471	1				
M0	19	2.847			1.62–5.00	<0.001	19	2.192	1.33–3.63			0.002		11	2.641	1.33–5.24			0.006
Multivariable model^b^																			
*MEF2C* Expression																			
Low (Q1–3)	477	1					477	1						371	1				
High (Q4)	152	0.986			0.72–1.36	0.929	152	1.135	0.86–1.49			0.365		101	1.324	0.91–1.92			0.137
Disease risk^a^																			
Standard-risk	292	1					292	1						213	1				
Low-risk	252	0.330			0.23–0.48	<0.001	252	0.419	0.31–0.56			<0.001		210	0.422	0.30–0.59			<0.001
High-risk	85	1.251			0.85–1.78	0.214	85	1.275	0.90–1.75			0.180		49	0.872	0.56–1.36			0.549
FAB Class																			
Not M0	610	1					610	1						461	1				
M0	19	1.981			1.09–3.59	0.024	19	2.382	1.05–5.42			0.038		11	1.646	0.78–3.45			0.187

^a^See “Methods” section for definition of cytogenetic/molecular disease risk

^b^Models were also adjusted for treatment arm, FAB category (M0 vs. no-M0), and age

## Discussion

Recent studies have highlighted a possible role of *MEF2C* in the molecular pathogenesis and therapy response of AML [3]. Using over 750 pretreatment bone marrow specimens from pediatric patients enrolled in a recent cooperative group phase 3 trial, ours is the first study to quantify *MEF2C* mRNA abundance by RT-PCR and comprehensively examine the relationship between *MEF2C* expression and disease characteristics as well as treatment outcome in pediatric AML. The findings from these investigations support three main conclusions. First, *MEF2C* is widely expressed in pediatric AML, with relative levels that vary considerably (>3000-fold) across bone marrows of patients with active disease. Second, high *MEF2C* expression is associated with adverse treatment outcome in pediatric AML. Specifically, in our cohort, patients with the highest relative *MEF2C* expression (4th quartile) less likely achieved a CR after one course of chemotherapy than the other patients; they also had an inferior OS and EFS and higher RR than patients within the lower 3 quartiles of *MEF2C* expression. And third, high *MEF2C* expression is associated with several adverse-risk features. Specifically, in participants of AAML0531, high relative expression of *MEF2C* was associated with a lower prevalence of cytogenetically/molecularly defined *low-risk* disease and higher prevalence of *standard-risk* disease, largely because of a lower prevalence of CBF leukemias or mutations in *NPM1* or *CEBPA* and a higher prevalence of leukemias with monosomy 7 or abnormalities involving 11q23. Conversely, high relative expression of *MEF2C* was associated with some better risk features, particularly a lower prevalence of *FLT3/ITD* (10 vs. 18 %; Table 2). Still, the associations between adverse cytogenetic or molecular disease risk features with high *MEF2C* expression dominated and largely accounted for the association between *MEF2C* expression and outcome. In fact, after multivariable adjustment, *MEF2C* expression was not apparently associated with outcome. As *MEF2C* expression does not provide prognostic information that is independent of established risk factors, *MEF2C* may not be particularly useful as a response biomarker. Nonetheless, high *MEF2C* expression was found to be associated with inferior efficacy of curative-intent, intensive AML chemotherapy. These data may, ultimately, provide a strong rationale for therapeutic targeting of *MEF2C* transcriptional activation in this disease.

Because of the genetic, molecular, and immunophenotypic heterogeneity of human AML, identification of pharmacologic drugs suitable for reasonably large subsets of patients has remained challenging. Therefore, unraveling signaling aberrancies shared by many of the leukemias could be useful for the development of risk-directed, mechanism-based therapies. Our data suggest the possibility that targeting *MEF2C*-induced signaling could serve as one such strategy. Very recent studies have identified *MEF2C* as a key factor in regulating *suppressor of cytokine signaling-2* (*SOCS2*) in normal and malignant hematopoiesis and indicated that the *MEF2C*/*SOCS2* regulatory network might confer leukemic stemness features to a neoplastic hematopoietic clone [25]. Consistent with a close relationship between *MEF2C* and *SOCS2*, we [26] and subsequently others [25] have provided evidence that high *SOCS2* expression is associated with poor survival in AML. Studies in T-ALL and colon cancer cells have indicated that *MEF2C* may inhibit BCL2-regulated apoptosis and can function as a regulator of cell proliferation [7, 27]. A similar mechanism of apoptosis resistance induced by *MEF2C* in AML cells may explain the apparent association between *MEF2C* overexpression and failure of AML chemotherapy. Further experimental studies will be required to elucidate the mechanisms of *MEF2C*-induced leukemogenesis and effective therapeutic strategies to block them.

It is a strength of our analysis that we included a large number of diagnostic specimens from patients treated homogeneously on a recent cooperative group trial, thereby increasing the precision of the outcome estimates. On the other hand, our studies have some limitations that need to be acknowledged. First, despite the use of over 750 specimens, our study was not large enough to allow for extensive multivariate adjustments. Because of the sample size of the individual risk groups, our ability to perform subset analyses was similarly limited. Second, since unsorted bone marrow specimens were used for our studies, differences in *MEF2C* abundance between specimens may not necessarily reflect differences in AML blasts but, rather, other (i.e., non-leukemic) cells or varying compositions of less mature and more mature AML cells. Gene expression studies in human material indicate that higher *MEF2C* mRNA levels are found in less mature hematopoietic cells, including LSC populations [28, 29]. Additional studies will be required for the identification of the exact cellular origins of the greatly variable amounts of *MEF2C* and more detailed analyses of relative expression levels along the cellular differentiation path of AML cells. Third, we only had cryopreserved specimens available for our analyses. Future studies will be necessary to determine to what degree, if any, *MEF2C* expression changes in the cryopreservation process. And fourth, we were unable to formally study whether high *MEF2C* mRNA expression leads to high MEF2C protein expression, a relationship that would provide a strong rationale for therapeutic targeting of MEF2C transcriptional activation in AML. However, preliminary data from ongoing laboratory studies indeed suggest that dysregulated *MEF2C* transcription results in MEF2C protein overexpression and confers enhanced AML cell survival (A. Kentsis, personal communication). If clinically exploitable strategies to counteract MEF2C signaling were developed, it is conceivable that *MEF2C* expression could become a biomarker of interest for successful drug development [30], e.g., to identify the subsets of patients most suitable for MEF2C-directed therapy.

## Conclusions

Our data indicate that high *MEF2C* expression identifies a subset of pediatric and adolescent AML patients with adverse-risk disease features and, consequently, significantly increased risk for primary treatment failure, relapse, and poor leukemia-free and overall survival. With confirmation that high *MEF2C* mRNA expression leads to overexpression of MEF2C protein, these findings provide the rationale for therapeutic targeting of MEF2C transcriptional activation in AML.

## Methods

### Patient samples

Cryopreserved pretreatment (“diagnostic”) specimens from patients enrolled in AAML0531 who consented to the biology studies and had bone marrow samples were available and were included in this study. The patient and disease (cytogenetic/molecular) characteristics of the subset of AAML0531 patients studied in this analysis were relatively comparable to patients not studied in this analysis. However, there were some differences in disease characteristics (i.e., higher proportion of patients with inv(16)/t(16;16) [*P* = 0.007] and low-risk disease [*P* < 0.001]) as well as better short-term outcomes (i.e., CR rate after one course of therapy [*P* = 0.005] albeit not rate of MRD [*P* = 0.132]), but OS was similar (*P* = 0.52) and EFS was only slightly better (*P* = 0.04).

### Risk stratification

A combination of cytogenetic and molecular abnormalities was used to stratify participants into risk groups. A patient was considered *low-risk* if a chromosomal abnormality/mutation was present in core binding factors (CBF; t(8;21) or inv(16)/t(16;16)), *nucleophosmin* (*NPM1*) (unless a *FLT3/internal tandem duplication* (ITD) mutation with high allelic ratio [≥0.4] was also present), or *CEBPA*; for *CEBPA*, both single and double mutations were considered favorable [31]. Patients were classified as *high-risk* if they had monosomy 5 or deletion of 5q (−5/5q−), monosomy 7 (−7), or *FLT3/ITD* with high allelic ratio (0.4 or higher). All other patients with data sufficient for classification were considered *standard-risk*.

### Detection and quantification of minimal residual disease (MRD)

Residual AML was quantified in bone marrow aspirates collected at the end of the first induction course by multiparameter flow cytometry using a “different-from-normal” approach as previously described [32].

### Quantification of *MEF2C* expression in unsorted AML specimens

Total RNA from unsorted diagnostic AML specimens was extracted with the AllPrep DNA/RNA Mini Kit using the QIAcube automated system (Qiagen, Valencia, CA). After quantification with a microvolume spectrophotometer (NanoDrop; Thermo Scientific, Wilmington, DE), 10 ng of total RNA was subjected to quantitative reverse-transcriptase polymerase chain reaction (qRT-PCR; 7900 Real-Time PCR System; Applied Biosystems; Foster City, CA) using TaqMan primers per manufacturer’s instructions to determine expression of *MEF2C* and, for normalization, the housekeeping gene, *GUSB*. Primer probe sets were as follows: *MEF2C* was designed to amplify sequence at the junction of exons 6 and 7, and *GUSB* was designed to amplify sequence at the junction of exons 8 and 9 (Hs00231149_m1 and Hs00939627_m1, respectively; Applied Biosystems). Patient samples were run in duplicate, and the ΔΔCT method quantified as 2^(−ΔΔCT)^ [33, 34] was used to determine the expression levels of *MEF2C* relative to *GUSB*.

### Statistical analysis

Data from AAML0531 were current as of December 31, 2013. The median (range) of follow-up for patients alive at last contact was 4.3 (0.02–7.1) years. The Kaplan-Meier method [35] was used to estimate OS (defined as time from study entry to death) and EFS (time from study entry until failure to achieve CR during induction, relapse, or death). RR was calculated by cumulative incidence methods defined as time from the end of induction I for patients in CR to relapse or death where deaths without a relapse were considered competing events [36]. Patients who withdrew from therapy due to relapse, persistent central nervous system (CNS) disease, or refractory disease with >20 % bone marrow blasts by the end of induction I were defined as induction I failures. The significance of predictor variables was tested with the log-rank statistic for OS and EFS and with Gray’s statistic for RR. All estimates are reported with two times the Greenwood standard errors. Children lost to follow-up were censored at their date of last known contact. Cox proportional hazards models [37] were used to estimate the hazard ratio (HR) for defined groups of patients in univariate and multivariate analyses of OS and EFS. Analyses of univariable OS for low-risk patients and multivariable EFS for all patients violated the proportional hazards assumption, and therefore, a parametric cure regression model was used to estimate the HR. Competing risk regression models were used to estimate HRs for univariate and multivariate analyses of RR. The chi-square test was used to test the significance of observed differences in proportions, and Fisher’s exact test was used when data were sparse. Differences in medians were compared by the Mann-Whitney or Wilcoxon signed-rank tests as appropriate. A *P* value <0.05 was considered statistically significant.

## Ethics, consent, and permissions

Informed consent was obtained from all study subjects in accordance with the Declaration of Helsinki, and the institutional review boards (IRBs) of all participating institutions approved the clinical protocol. IRB approval was obtained from Fred Hutchinson Cancer Research Center before conduct of this biological study, which was also approved by the COG Myeloid Disease Biology Committee and the National Cancer Institute Cancer Therapy Evaluation Program.
