# Erratum to: High expression of myocyte enhancer factor 2C (MEF2C) is associated with adverse-risk features and poor outcome in pediatric acute myeloid leukemia: a report from the Children’s Oncology Group

**DOI:** 10.1186/s13045-016-0364-0

**Published:** 2016-11-30

**Authors:** George S. Laszlo, Todd A. Alonzo, Chelsea J. Gudgeon, Kimberly H. Harrington, Alex Kentsis, Robert B. Gerbing, Yi-Cheng Wang, Rhonda E. Ries, Susana C. Raimondi, Betsy A. Hirsch, Alan S. Gamis, Soheil Meshinchi, Roland B. Walter

**Affiliations:** 1 Clinical Research Division, Fred Hutchinson Cancer Research Center, 1100 Fairview Ave N, D2-190, Seattle, WA 98109-1024 USA; 2 Department of Biostatistics, University of Southern California, Los Angeles, CA USA; 3 Children’s Oncology Group, Monrovia, CA USA; 4 Molecular Pharmacology and Chemistry Program, Sloan Kettering Institute, New York, NY USA; 5 Department of Pediatrics, Memorial Sloan Kettering Cancer Center, New York, NY USA; 6 Weill Medical College of Cornell University, New York, NY USA; 7 Department of Pathology, St. Jude Children’s Research Hospital, Memphis, TN USA; 8 Department of Laboratory Medicine and Pathology, University of Minnesota Cancer Center, Minneapolis, MN USA; 9 Division of Hematology-Oncology, Children’s Mercy Hospitals and Clinics, Kansas City, MO USA; 10 Department of Pediatrics, University of Washington, Seattle, WA USA; 11 Department of Medicine, Division of Hematology, University of Washington, Seattle, WA USA; 12 Department of Epidemiology, University of Washington, Seattle, WA USA

## Erratum

The original article [1] contains an omission of information in the **Financial support** sub-section of the **Declarations** section.

The authors would like to acknowledge the Andrew McDonough B+ Foundation for financial support of co-author Dr Yi-Cheng Wang throughout this study, and regret this inadvertent omission from the original publication.

## References

[1] Laszlo GS (2015). High expression of myocyte enhancer factor 2C (MEF2C) is associated with adverse-risk features and poor outcome in pediatric acute myeloid leukemia: a report from the Children’s Oncology Group. J Hematol Oncol.

